# Function matters: Coronavirus cross-binding antibodies do not cross-neutralize

**DOI:** 10.3389/fmed.2022.924426

**Published:** 2022-08-02

**Authors:** Maria R. Farcet, Julia Schwaiger, Michael Karbiener, Thomas R. Kreil

**Affiliations:** Global Pathogen Safety, Takeda Manufacturing Austria AG, Vienna, Austria

**Keywords:** immune deficiency, neutralizing antibodies, COVID-19, intravenous immunoglobulin, immunoglobulin, plasma, hyperimmune globulin, coronaviruses (CoVs)

## Abstract

**Background:**

During the current pandemic, the severe acute respiratory syndrome coronavirus-2 (SARS-CoV-2) neutralization capacity of the immunoglobulin (IG) supply has changed from undetectable for lots manufactured from plasma collected before the pandemic, to now highly potent.

**Objective:**

As antibodies induced by exposure to or vaccination against coronaviruses were shown to be cross-coronavirus reactive, it was of interest to understand whether SARS-CoV-2 neutralizing antibodies would result in increased functional IG potency also against seasonal coronaviruses.

**Methods:**

IG lots from US plasma collected before SARS-CoV-2 emerged and collected during the pandemic were analyzed by live virus neutralization assay for SARS-CoV-2 and seasonal human coronaviruses (HCoVs) NL63 and OC43 neutralizing antibody content.

**Results:**

Pre-pandemic IG showed no SARS-CoV-2 neutralizing antibody titers. However, IG lots produced from plasma of post-coronavirus disease 2019 (COVID-19) individuals exhibited robust anti-SARS-CoV-2 potency (1,267 IU/ml) which further increased ~4-fold in pandemic IG lots reaching a mean titer of 5,122 IU/ml. Nonetheless, neutralizing antibody potencies to the HCoVs NL63 and OC43 remained stable over this period, i.e., have not increased correspondingly.

**Conclusion:**

The present results show that cross-coronavirus-reactive antibodies are not cross-neutralizing, i.e., SARS-CoV-2 antibodies do not neutralize seasonal coronaviruses NL63 and OC43.

## Introduction

Antibodies induced by exposure to or vaccination against coronaviruses were shown to be cross-coronavirus reactive by binding and pseudovirus neutralization assays (1, 2), also against the more recently emerged and now global severe acute respiratory syndrome coronavirus-2 (SARS-CoV-2). In contrast, concentrated immunoglobulin (IG) preparations manufactured from plasma collected from many thousand donors before the coronavirus disease 2019 (COVID-19) pandemic did not cross-neutralize SARS-CoV-2 in gold standard live virus neutralization assays (3, 4).

While antibody binding and pseudovirus neutralization assays are more convenient to conduct as they do not require work with an infectious virus in a biocontainment laboratory infrastructure to generate biomarker and immune correlation data, the functional basis for antibody-mediated protection is virus neutralization. And while the medical relevance of antibody binding assays is very different from the demonstration of antibody-mediated neutralization of virus infectivity, these specific technical terms are not always used quite so adequately, with results of binding assays occasionally referred to as “neutralizing” (5).

Recently, the immune response to SARS-CoV-2 was found to also expand pre-existing cross-binding antibodies to seasonal coronaviruses (6, 7). Whether or not the observation also translates into cross-neutralization of seasonal and the currently pandemic coronavirus has, however, not yet been investigated by functionally more relevant virus neutralization assays. More generally, while neutralizing antibodies (nAbs) are now understood to be highly predictive of immune protection from SARS-CoV-2 symptomatic infection (8), any potential cross-neutralization of seasonal and the now pandemic coronaviruses has not yet been investigated.

To examine whether infection by or vaccination against SARS-CoV-2 would also induce or increase cross-neutralization of seasonal coronaviruses, antibody concentrates manufactured by a licensed manufacturing process (Gammagard Liquid, Baxalta US Inc.) from either SARS-CoV-2 naïve plasma collected before the COVID-19 pandemic (pre-pandemic), from plasma of serologically confirmed post-COVID-19 donors (post-COVID), or from plasma collected after effective vaccination campaigns had reached a dominant proportion of plasma donors (pandemic) were tested for neutralization of SARS-CoV-2, as well as the human coronavirus (HCoV)-OC43, another beta coronavirus, or HCoV-NL63, an alpha coronavirus that uses the same cellular receptor for infection as SARS-CoV-2, in live virus neutralization assays (9, 10).

## Methods

### Immunoglobulin preparations

A total of 363 immunoglobulin (IG) lots (Gammagard Liquid; Baxalta US Inc., Lexington, MA or manufactured using the same process) were characterized for SARS-CoV-2 and HCoV neutralizing antibody (nAb) content. Of these, the nAb content of 16 pre-pandemic, 21 post-COVID and 18 SARS-CoV-2 high tittered pandemic IG lots were directly compared against each other. The pre-pandemic and pandemic IG lots were fractionated from US plasma collected by plasmapheresis and released between April 2020 and June 2020 [due to the delay between plasma donation and completion of IG manufacture; similar to (3)] and between July 2021 and September 2021 [similar to (11)], respectively. Post-COVID IGs were manufactured exclusively from post-COVID-19 plasma collected in the US or Austria, as a potential hyperimmune treatment for COVID-19 [CoVIg-19, (12)], also studied in a phase three clinical trial [ClinicalTrials.gov: NCT04546581; (13)].

For investigation of coronavirus titer changes throughout the pandemic, 326 IG lots (including the 18 SARS-CoV-2 high titered pandemic lots), released between September 2020 and October 2021 (13–31 lots per month), were tested.

### Measurement of SARS-CoV-2 neutralizing antibodies

SARS-CoV-2 neutralizing antibody titers were determined as previously reported (14). A fully validated analytical method was used and the National Institute of Biological Standards and Control (NIBSC, Potters Bar, UK) WHO International Standard 20/136, for which a potency in international units have been assigned (15, 16), was included in the study and the concentration of SARS-CoV-2 neutralizing antibodies, therefore, reported in IU/ml.

### Measurement of HCoV neutralizing antibodies

HCoV-NL63 and HCoV-OC43 neutralizing antibody titers were determined similarly as previously reported (14). In short, 2-fold serially diluted samples were incubated with the same volumes of HCoV-NL63 (provided by Lia van der Hoek, University of Amsterdam, the Netherlands) or HCoV-OC43 (American Type Culture Collection [ATCC] VR-1558) at 10^3.0^ tissue culture infectious doses 50% per milliliter (TCID_50_/ml). After incubation for 150 min the mixtures were incubated on LLC-MK2 cells (for HCoV-NL63; ATCC CCL-7) or MRC-5 cells (for HCoV-OC43; ATCC CCL-171) in 8-fold replicates per dilution. The virus-induced cytopathic effect was analyzed after 9–11 (HCoV-NL63) or 6–8 (HCoV-OC43) days of incubation, respectively. The reciprocal test article dilution resulting in 50% virus neutralization (μNT_50_) was determined using the Spearman-Kärber formula. The calculated neutralization titer for 50% of the wells was normalized to an internal assay control [IG lot manufactured from pre-pandemic plasma; conceptually identical to (3)]: for this internal assay control, a “reference μNT_50_ titer” (determined from 10 independent assays) was defined and for each assay this internal assay control was tested in parallel to the samples. The μNT_50_ titer of each sample was normalized to the μNT_50_ titer of the internal assay control from the same assay and the “reference μNT_50_ titer.” The resulting value was designated μNT_50_ titer [norm. 1:X]. The controlled assays included several validity criteria, i.e., confirmatory titration of input virus infectivity and cell viability.

### Graphs and statistical analysis

Data analysis and visualization were done using GraphPad Prism v8.1.1 (San Diego, CA) and R Studio v1.1.383 (Boston, MA). Descriptive statistics of IGs allocated to the three groups pre-pandemic, post-COVID, and pandemic were realized as box plots showing medians with 25th and 75th percentiles and whiskers depicting minimum and maximum values. For the temporal development of coronavirus-neutralizing potency, IG lots were grouped according to release month and for each group, the geometric mean neutralizing potency ±95% confidence interval was calculated.

## Results

### Effects of COVID-19 infection and COVID-19 vaccinations on coronavirus neutralization capacity of immunoglobulin

**Figure 1:**
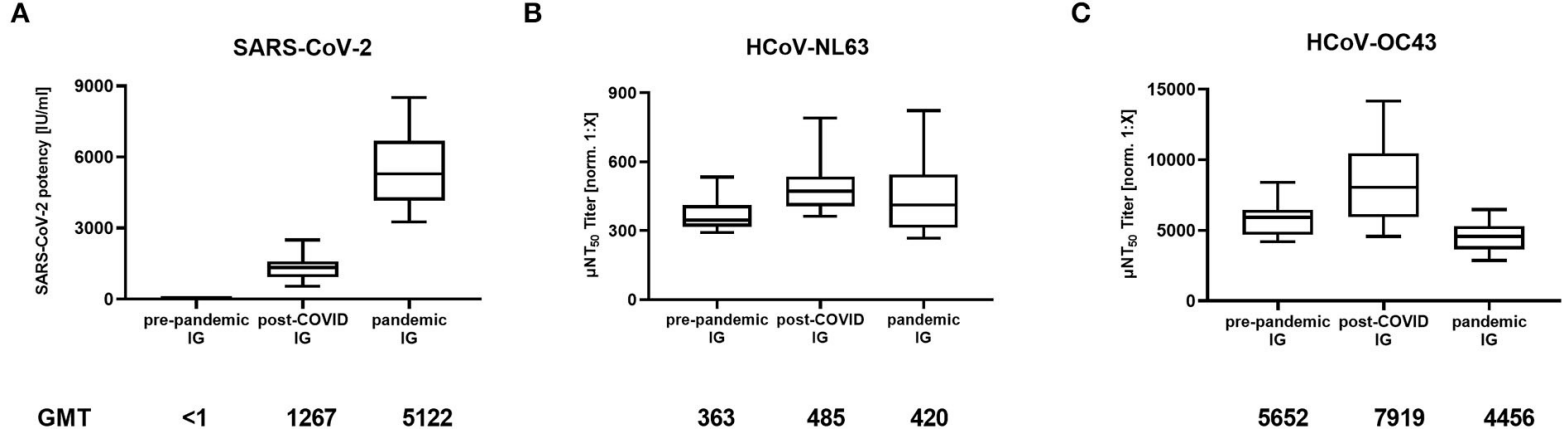
Neutralizing antibody titers in immunoglobulin (IG) lots manufactured from pre-pandemic plasma (*N* = 16), plasma of post-COVID-19 individuals (post-COVID, *N* = 21), and plasma of mostly COVID-19 vaccinated donors (pandemic, *N* = 18) against **(A)** SARS-CoV-2, **(B)** Human Coronavirus NL63 (HCoV-NL63) and **(C)** HCoV-OC43. Box plots show medians with 25th to 75th percentile ± min/max as whiskers. Geometric mean titers (GMT) are indicated below the x-axis.

IG lots produced from pre-pandemic plasma (*N* = 16) did not neutralize SARS-CoV-2 (Figure 1A). Post-COVID lots, manufactured from plasma collected after donors had recovered from COVID-19 (*N* = 21), showed an average SARS-CoV-2 neutralization activity of 1,267 IU/ml (geometric mean titer, GMT, min: 536 IU/mL, max: 2,503 IU/mL), which further increased to 5,122 IU/ml (min: 3,256 IU/mL, max: 8,505 IU/mL) in SARS-CoV-2 high titered pandemic IG preparations produced from a predominantly vaccinated plasma donor population (*N* = 18). As expected, the pre-pandemic IG preparations provided for potent neutralization of HCoV-NL63 (363 μNT_50_ [norm. 1:X], GMT, min: 291 μNT_50_ [norm. 1:X], max: 533 μNT_50_ [norm. 1:X]; Figure 1B) as well as HCoV-OC43 (5,652 μNT_50_ [norm. 1:X], GMT, min: 4,196 μNT_50_ [norm. 1:X], max: 8,393 μNT_50_ [norm. 1:X]; Figure 1C). In contrast to SARS-CoV-2, the neutralization capacity of HCoV-NL63 and HCoV-OC43 was largely comparable between pre-pandemic, post-COVID, and pandemic IG lots (Figure 1), i.e., titers were within only one of the 2-fold dilution steps as used in the assays and thus within the variability of such functional assay systems.

### Development of anti-coronavirus potencies during the COVID-19 pandemic

**Figure 2:**
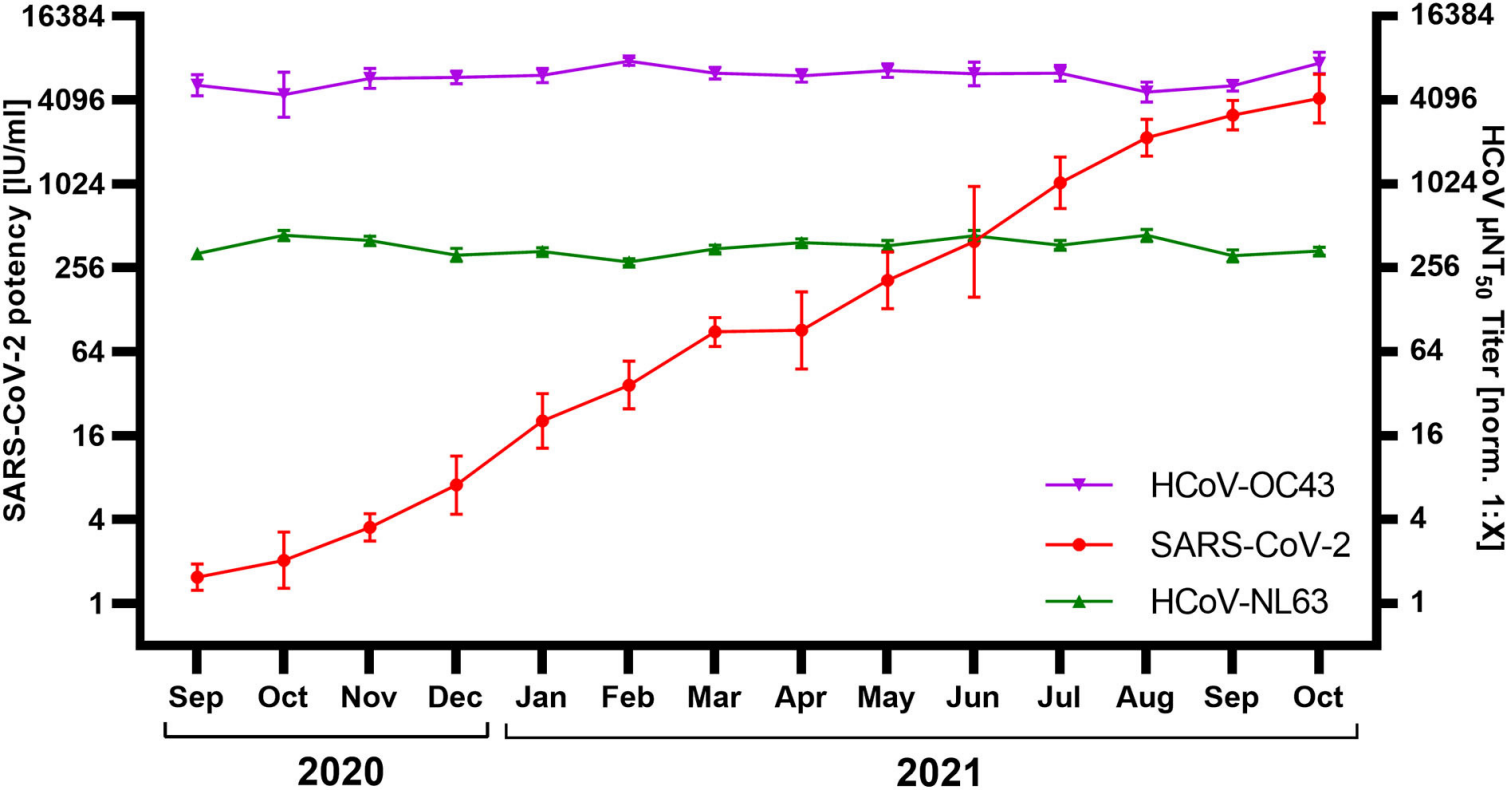
Neutralization of SARS-CoV-2, HCoV-NL63, and HCoV-OC43 by immunoglobulin (IG) released September 2020–October 2021 (*N* = 326; 13–31 lots/month). SARS-CoV-2 neutralization was normalized to WHO International Standard 20/136 and reported as international units / milliliter (IU/ml). HCoV titers were normalized to an internal standard and reported as μNT_50_ titer [norm. 1:X]. Shown are geometric mean titer ± 95% confidence intervals.

326 IG lots released between September 2020 and October 2021 were screened for SARS-CoV-2 and HCoV nAbs. IG lots released in September 2020 showed an average SARS-CoV-2 neutralization activity of 1.5 IU/ml (GMT; *N* = 26; lower/upper 95% CI: 1.2/1.9 IU/mL) which increased steadily to 4,210 IU/ml (*N* = 28; lower/upper 95% CI: 2,817/6,291 IU/mL) in October 2021 (Figure 2). However, HCoV titers remained stable over this period. A comparison of the monthly GMT showed a range of 282–438 μNT_50_ [norm. 1:X] for HCoV-NL63 and a range of 4,481–7,790 μNT_50_ [norm. 1:X] for HCoV-OC43 throughout the whole period surveyed.

## Discussion

Collectively, the two orthogonal analyses described above (stratification of IG according to SARS-CoV-2 status of plasma donors; temporal development of IG neutralization capacity) corroborate that results obtained from antibody binding and pseudovirus neutralization assays (6, 7) do not necessarily translate to live virus neutralization. As previously reported (3), no SARS-CoV-2 nAbs were detected in pre-pandemic IG lots. However, post-COVID IG lots exhibited potent neutralization activity, and pandemic IG preparations from a mostly vaccinated plasma donor population were even ~4-fold more potent, entirely consistent with an earlier report (11). In contrast, the pre-pandemic IG preparations provided for potent neutralization of HCoV-NL63 as well as HCoV-OC43, consistent with the relatively wide circulation of these seasonal coronaviruses and the use of several thousand plasma donations in the preparation of each IG lot. However, while all the post-COVID IG lots provided for highly effective neutralization of SARS-CoV-2, in contrast to the non-neutralizing IG lots from pre-pandemic plasma, their neutralization capacity of HCoV-NL63 and HCoV-OC43 was quite similar to the IG lots from pre-pandemic plasma. The same was observed for the pandemic lots of IG, with a ~4-fold more potent SARS-CoV-2 neutralization capacity yet again a similar neutralization capacity for the two seasonal coronaviruses tested.

In contrast, a recent publication reported a ~4-fold difference in HCoV-OC43 neutralizing antibodies between paired pre- and post-COVID-19 and COVID-vaccinated plasma samples, and passive transfer of plasma from vaccinated individuals even conferred some protection against HCoV-OC43 in a mouse model of infection (7). These results obtained from a more limited number of plasma samples may appear to be different from the data of several hundred IG lots, each reflective of the serostatus of several thousand plasma donors, as presented here.

The potency of SARS-CoV-2 neutralization in plasma is, however, particularly early after infection or vaccination, mediated by immunoglobulins of the IgM as well as IgG isotypes, whereas the commercial manufacturing process of the IG preparations concentrates only IgG into final products. Conceivably thus, IgM may be more coronavirus cross-reactive and even cross-neutralizing as compared to IgG after affinity maturation, a plausible explanation for the different findings. Future observational studies are expected to explore how well the anti-SARS-CoV-2 potency of currently manufactured IG preparations translates into protection from symptomatic COVID-19.

During the course of the current pandemic, the SARS-CoV-2 neutralization capacity of the IG supply manufactured from plasma collected in the US has changed from undetectable for lots manufactured from plasma collected before the pandemic (3), to rapidly increasing based on an increasing percentage of post-COVID-19 plasma donors (14), to now highly potent (11), based on vaccination against COVID-19 which, at least for the widely used mRNA vaccines, induces antibody titers even higher than post-COVID-19 (17). If infection by or vaccination against SARS-CoV-2 were to also induce cross-coronavirus neutralizing IgG antibodies, these should increase markedly between IG lots produced from plasma collected before and then across the evolution of the pandemic. This was, however, not the case. Neutralization of HCoV-NL63 and HCoV-OC43 by IG lots remained stable over the period during which SARS-CoV-2 neutralization titers increased from initially non-detectable to now ~2,000-fold higher than the limit of detection of the functional assay used.

As a limitation of our study, the investigated IG lots did not contain plasma from post-COVID-19 individuals which had been infected by the currently dominant Omicron variant of SARS-CoV-2, or the preceding Delta variant. However, the main anti-SARS-CoV-2 potency in IG lots was raised through COVID-19 vaccination rather than SARS-CoV-2 infection (11), and a lack of cross-neutralization by these antibodies against the seasonal coronaviruses was shown in our study.

In summary, IgG antibodies against seasonal and the currently pandemic coronavirus can be cross-reactive (6, 7), but likely they do not cross-neutralize these different coronaviruses. The finding is of significant clinical relevance as people with immune deficiency (PID) even under IG substitution therapy may still experience virus breakthrough infections. A recent study has found that in 56% of respiratory exacerbations in PIDs, a pathogenic virus was identified, of which 23% were seasonal coronaviruses (18).

Given the now highly potent SARS-CoV-2 neutralization in IG lots from plasma collected post-COVID-19 or after highly effective vaccination against SARS-CoV-2 infection, and the demonstration of cross-reaction and even boosting of cross-coronavirus binding antibodies (6), it may be tempting to speculate about improved protection of PID patients also against seasonal coronaviruses by IG treatment in general. However, while the beneficial effects of the antibody response to infection might be multi-faceted, it is the levels of neutralizing antibodies that were shown to predict immune protection from SARS-CoV-2 infection (8). Thus, although cellular immune responses provide an additional mechanism against virus infection, the absence of cross-coronavirus neutralization by antibodies would argue for a more cautious interpretation.

## Data availability statement

The raw data supporting the conclusions of this article will be made available by the authors, without undue reservation.

## Ethics statement

Ethical review and approval was not required for the study that analyzed biological material pooled from thousands of humans, in accordance with the local legislation and institutional requirements. Written informed consent for participation was not required for this study in accordance with the national legislation and the institutional requirements.

## Author contributions

MF and TRK designed the study. JS and MK conducted data analysis and prepared a graphical representation of the data. All authors drafted the manuscript and approved the submitted version.

## Funding

This work was supported by Takeda Manufacturing Austria AG.

## Conflict of interest

The authors are employees of Takeda Manufacturing Austria AG, Vienna, Austria. MF, MK, and TRK have Takeda stock interest.

## Publisher's note

All claims expressed in this article are solely those of the authors and do not necessarily represent those of their affiliated organizations, or those of the publisher, the editors and the reviewers. Any product that may be evaluated in this article, or claim that may be made by its manufacturer, is not guaranteed or endorsed by the publisher.
